# Comparative assessment for hyperaccumulatory and phytoremediation capability of three wild weeds

**DOI:** 10.1007/s13205-014-0194-0

**Published:** 2014-01-19

**Authors:** Madhuri Girdhar, Neeta Raj Sharma, Hasibur Rehman, Anupam Kumar, Anand Mohan

**Affiliations:** 1 Department of Biotechnology, Lovely Professional University, Chehru, Phagwara, India; 2 Department of Biology, Faculty of Sciences, University of Tabuk, Tabuk, Saudi Arabia

**Keywords:** *Cannabis sativa*, *Solanum nigrum*, *Rorippa globosa*, Hyperaccumulation, Optimum, Phytoremediation, Phytochelates, Intrinsic capacity

## Abstract

The composition and the organization of soil are changing rapidly by the diverged mankind activities, leading to the contamination of environment. Several methods are employed to clean up the environment from these kinds of contaminants, but most of them are costly and ineffective to yield optimum results. Phytoremediation is a natural green technology, which is eco-friendly for the removal of toxic metals from the polluted environment. Phytoremediation is a cost-effective technique through which the cleanup of contaminated soil laced with heavy metals is performed by wild weeds and small herbal plants. The phytoremediation technique provides a promising tool for hyperaccumulation of heavy metals; arsenic, lead, mercury, copper, chromium, and nickel, etc., by the wild weeds and that has been discussed here in detail in case of *Cannabissativa*, *Solanum nigrum* and *Rorippa globosa*. In general, weeds that have the intrinsic capacity to accumulate metals into their shoots and roots, have the ability to form phytochelates and formation of stable compound with ions. This behavior of accumulation along with chelate and stable compound formation is utilized as a tool for phytoremediation activity.

## Introduction

Phytoremediation is technically a collection of plant-based technologies that cause remediation of environmental pollution exploiting capability of plants or wild weeds for the remediation of contaminated soil (Cunningham et al. 1997; Flathman and Lanza 1998). Metal hyper accumulating plants have gained increased attention because of their potential to accumulate heavy metals and have application in decontamination of metal polluted soil. Acting as an integrated multidisciplinary approach for the cleanup of contaminated soils, phytoremediation combines the disciplines of plant physiology, soil microbiology, and soil chemistry (Cunningham and Ow 1996). The goal of phytoremediation is the removal of toxic metals from the soil (Reeves and Baker 2000). This current review provides the information about the various eco-friendly techniques and technologies implying to the phytoremediation process and the hyperaccumulating nature of various wild weeds (*Cannabis sativa*, *Solanum nigrum* and *Rorippa globosa*), which have enough capability of heavy metal accumulation. Wild weeds are suitable for this purpose because of their inherent resistant capability and their non-suitability for fodder purpose. Metals are required for a variety of metabolic processes in all organisms; however, because many metals can be toxic, plants have evolved systems to regulate the uptake and distribution of metals. The uptake of metal occurs primarily through the roots of plants, therefore this becomes the primary site for regulating their accumulation. In extreme conditions, where the metal concentration is very high in soil; leaves, roots and shoots play an important role by accumulating the metal more than required by their physiology. From various studies, it has been shown that metal concentration in leaves is >0.1 mg/g dry weight for cadmium (Cd) metal and >1 mg/g dry weight for lead, copper, and nickel metal (Reeves and Baker 2000). Soil chemistry and soil microbiology, which is contaminated by heavy metals can be cleaned up and maintained by these accumulatory activities of such plants (Cunningham and Ow 1996). Tissues of higher plants accumulate a very high concentration of metals without showing toxicity (Klassen et al. 2000; Bennett et al. 2003).

**Fig. 1:**
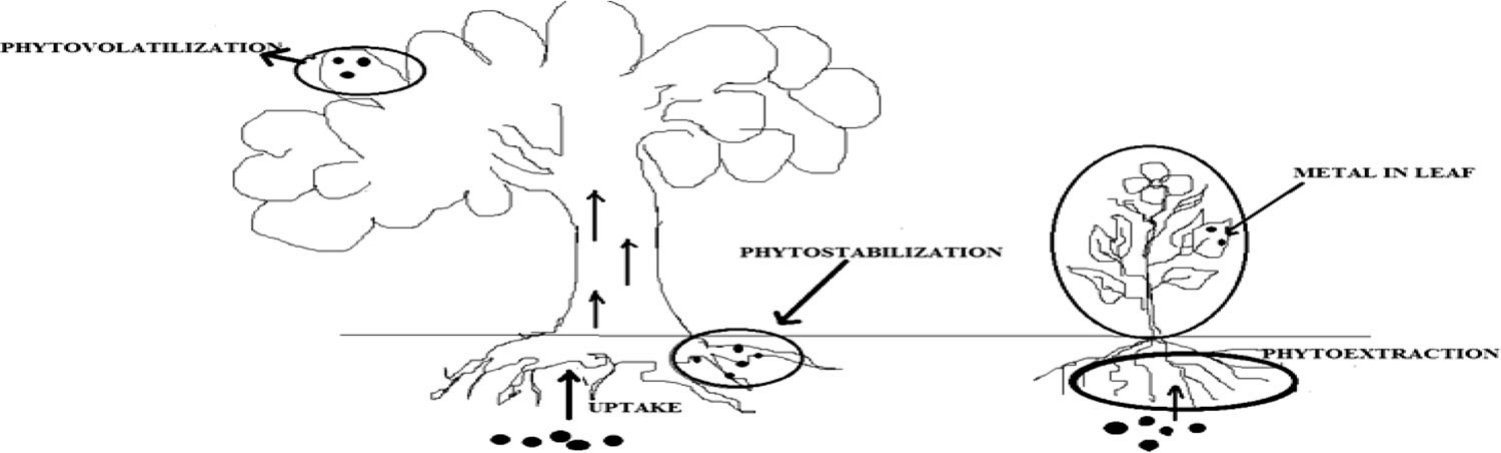
Different types of phytoremediation processes adapted from Singh et al. (2003), Suresh and Ravishankar (2004)

There are various mechanisms associated with phytoremediation, phytoextraction, phytovolatization, rhizofiltration, and phytodegradation (Fig. 1). Phytoextraction is a mechanism in which plant roots absorb contaminated ground water and then transport it from roots to various parts of the plant (Salt et al. 1998). The cost involved in the phytoextraction as compared with the conventional soil remediation technique, is tenfolds less per hectare. It means phytoextraction is a cost-effective technique (Salt et al. 1995). The development of phytoextraction technique comes from the discovery of variety of wild weeds, often endemic to naturally mineralized soils that concentrate high amount of essential and non-essential heavy metals. In the same context, *R. globosa* shows Cd hyperaccumulation up to certain extent as shown in the work of Yuebing et al. (2007).

Rhizofiltration is a cost-competitive technology in the treatment of surface water or groundwater containing low but significant concentrations of heavy metals such as Cr, Pb, and Zn (Ensley 2000). Rhizofiltration can be used for metals (Pb, Cd, Cu, Ni, and Cr) that are retained only within the roots. It is a phytoremediative technique designed for the removal of metals in aquatic environments. Hydroponic technique is being used in which the plants first grow in nutrient medium and then they are transferred to the metal polluted sites where the plants accumulate and concentrate the metals in their various body parts, especially roots (Flathman and Lanza 1998; Salt et al. 1995; Dushenkov et al. 1995; Zhu et al. 1999b) (Table 1).

**Table 1:** Few natural plant metal-hyperaccumulator species and their bioaccumulation potential

Metals	Plant species	Amount [gm/kg (d.m.)]	References
Cd	*Rorippa globosa*	>1	Sun et al. (2007)
Se	*Brassica juncea*	2.0	Orser et al. (1999)
Cr	*Salsola kali*	2.9	Gardea-Torresday et al. (2005)
Pb	*Thlaspi rotundifolium*	0.13–8.2	Reeves and Brooks (1983)
Cd	*Thlaspi caerulescens*	10.0	Lombi et al. (2001)
Ni	*Alyssum bertolonii*	>10.0	Morrison et al. (1980)
Co	*Haumaniastrum robertii*	10.2	Brooks (1977)
Cu	*Ipomea*	12.3	Baker and Walker (1990)
Mn	*Phytolacca acinosa*	19.3	Xue et al. (2004)
As	*Pteris vittata*	22.6	Ma et al. (2001)
Zn	*Thlaspi caerulescens*	30.0	Baker and Walker (1990)
Zn	*Chenopodium album* L.	33.5	Malik et al. (2010)
Pb	*Amaranthus viridis* L.	>43.0	Malik et al. (2010)
Cu	*Parthenium hysterophorus* L.	59.3	Malik et al. (2010)

Phytovolatization is a technique in which metals from the soil are taken up by the plant roots and by the process of transpiration they are released in the environment. This process works only when the metals are volatile in nature (Hg and Se) (USPA 2000). Apart from metals this has been established in case of organic contaminants also, as in the example of Poplar tree (*Liriodendron*) which is a phytovolatizer, and volatilizes up to 90 % of the trichloroethane absorbed from contaminated soil (McGrath and Zhao 2003).

Phytodegradation which is also known as phytotransformation is a process in which, the breakdown of contaminants occurs by plants through metabolic processes within the plant, or in the close surrounding of the plant through plant root symbiotic associations (McGrath and Zhao 2003). A metabolic process known as ex- planta occurs in which organic compounds are hydrolyzed into smaller units that can be absorbed by the plants. Some of these contaminants can be absorbed by the plant and are then broken down by plant enzymes. For the growth of the plant, these smaller pollutant molecules can be used as secondary metabolites for the growth of the plant (Prasad 1995).

### Mechanism of heavy metal uptake by plants

There are certain works that have shown the hyperaccumulatory action of *C. sativa* on Cd-contaminated soil. Some of the projects by the US Department of Energy have explored bioavailability of cadmium in the soil and have suggested that it depends on soil pH, redox potential, and rhizosphere chemistry. These factors determine the concentration of soluble Cd within the rhizosphere of the plant and the amount of Cd available for potential uptake by the plant. Soluble Cd could enter roots either by movement in the cell wall-free space (apoplastic pathway) or by transport across the plasma membrane (PM) of root cells and movement through the cytoplasm (symplastic pathway). The large membrane potential, which usually exists across the PM provides a driving force for the inward movement of Cd into cells. There are various types of channels that exist within the PM, which allow the Cd transport into the different parts of the plant. Secondary level of accumulation has been observed in stems and leaves with accumulated amount of Cd found to a lesser extent (Linger et al. 2005). The metals are absorbed from the soil into the roots and shoots by the process of translocation (phytoextraction). After uptake by roots, translocation into shoots is desirable because the harvest of root biomass is generally not feasible (US Department of Energy 1994). Plant uptake–translocation mechanisms are likely to be closely regulated. Plants generally do not accumulate elements beyond near-term metabolic needs, which are small ranging from 10 to 15 ppm of most trace elements, sufficient for most of the requirements (US Department of Energy 1994). Hyperaccumulator are exceptions and can accumulate toxic metals much beyond these limits up to the levels of thousands of ppm. With this capability of hyperaccumulation, these plants could successfully be used for phytoremediation purposes. During the process contamination is translocated from roots to shoots, which are harvested, causing contamination to be removed while leaving the original soil undisturbed (US Department of Energy 1994).

Hyperaccumulatory behavior and activity is dependent upon several physiological and biochemical rationales. Polychelatin formation is one of the important basic crucial factor for the hyperaccumulatory behavior. Polychelatins are the best-characterized heavy metals chelator in plants, especially in the context of Cd tolerance (Cobbett 2000). Hemp (*C. sativa*) roots demonstrated a strong resistance to heavy metals and have already shown hyperaccumulator-like potential (more than 100 mg/kg Cd in dry tissue), which more likely seems to depend on the plant development stage. High values of Cd accumulation achieved cannot be explained exclusively by passive ion uptake. Immobilization, by binding to the cell walls, is thought to play a minor role (Sanita di Toppi and Gabbrielli 1999). Poly Chelatins are known to be synthesized from glutathione (GSH) and its derivates by enzyme phytochelatin synthase in the presence of heavy metal ions (Cobbett 2000; Rea et al. 2004). The synthesis of polychelatin occurs in cytosol. With exposure of metal to the root of the plant, polychelatins coordinate to form ligand complexes with these metals, which are further sequestered into the vacuole. GSH also has a role in defense against heavy metals.

The other class of significantly notable chelating compound is metallothioneins. They are known to have a significant role in the detoxification of metals, and the induction of their synthesis in the plants occurs through exposure of root cells to heavy metals (Rauser 1999; Cobbett 2000; Clemens 2001; Hall 2002; Cobbett and Goldsbrough 2002; Rea et al. 2004). Metallothioneins (MTs) are sulfur-rich proteins of 60–80 amino acids and are known to contain 9–16 cysteine residues and are found in plants, animals and some prokaryotes (Rauser 1999; Cobbett 2000; Cobbett and Goldsbrough 2002). These cysteine-rich polypeptides exploit the property of heavy metals to bind to the thiol-groups of proteins and detoxify them. Other well-known property of MTs is to participate in Cu homeostasis (Cobbett and Goldsbrough 2002).

Polyamines are small and universal polycations involved in numerous processes of plant growth and development and have anti-senescence and anti-stress effects. These distressing effects are owned due to their acid neutralizing and antioxidant properties, along with their membrane and cell wall stabilizing abilities (Zhao and Yang 2008). Technically, polyamines strengthen the defense response of plants and modulate their activity against diverse environmental stresses including metal toxicity (Groppa et al. 2003), oxidative stress (Rider et al. 2007), drought (Yamaguchi et al. 2007), salinity (Duan et al. 2008) and chilling stress (Cuevas et al. 2008; Groppa and Benavides 2008). The accurate role of polyamines found in plants under metal stress has not been deduced yet. The most positive assumption regarding the functionality of polyamines is the protection of membrane systems and their stabilization from the toxic effects of metal ions, particularly the redox active metals. Spermine, spermidine, putrescine and cadaverine are some of the important polyamines, which have demonstrated the ability to scavenge free radicals in vitro (Drolet et al. 1986). Polyamines are also known to block the major vacuolar channels, the fast vacuolar cation channel. The accumulation of these vacuolar channels results in decreased ion conductance at the vacuolar membrane, which facilitates metal ion compartmentation (Brüggemann et al. 1998).

### *Cannabis sativa* general properties

*Cannabis* sativa is a flowering herb which is dioecious in nature. *Cannabis* is rich in cannabinoids which are psychoactive and physiologically active chemical compound produced by the dioecious flowers of the herb. *C. sativa* provides antispasmodic and muscle relaxant stimulating appetite (Zajicek et al. 2003). *C. sativa* exhibits a great diversity with its prominence in both wild and cultivated areas, and hence can be utilized for the phytoremediation purpose (Mura et al. 2004). Native to central and southern Asia, *Cannabis* prefers a warm and humid climate, but are very resilient and can live in many habitats, so long as the soil pH is between 5 and 7.

### Hyperaccumulative action by *Cannabis sativa*

Different studies carried out on *C. sativa* provide the leads that it can be used as an hyperaccumulator for different toxic trace metals such as lead, cadmium, magnesium, copper, chromium, and cobalt which pose a great risk to the ecological system. As already, it has been established that the sources of these polluting metals are various anthropogenic activities such as smelting, sewage sludge distribution and automobile emissions (Foy et al. 1978; Chronopoulos et al. 1997; Prasad and Hagemeyer 1999; Dahmani-Muller et al. 2000). Hyperaccumulator plants can be used to remediate the metal-contaminated soil from these anthropogenic activities. This technology implies on the above ground harvestable plant tissues that have the phytoaccumulation capacity in the roots of plants to absorb, translocate and concentrate heavy metals from contaminated soil. The wild species which are endemic to metalliferous soil accumulate a very high concentration of metal from the soil (Baker and Brooks 1989a, b). *C. sativa* is also known as industrial Hemp, because it has the capability of hyperaccumulation of industrial waste. The potential of Hemp crops is known to convert the wasteland into cultivated land, especially the area contaminated with heavy metal pollution (lead, copper, zinc, and cadmium) (Angelova et al. 2004). The Hemp is well suited for phytoremediation, and the fiber quality has not been negatively affected by uptake of metal.

Cd is known to be one of the most phytotoxic heavy metal (Salt et al. 1995; Prasad 1995). For the soil phytoremediation, a good alternative is provided by Hemp plant (*C. sativa* L.). Except for roots, the highest concentrations of metal are found in leaves, whereas the lowest are typically observed in seeds (Ivanova et al. 2003). The photosynthesis pathway is influenced in two ways by the cadmium metal: (1) Cadmium metal disturbs indirectly water and ion uptake by the plant which has a negative effect on the plant water status (Seregin and Ivanov 2001). (2) It directly affects the chloroplast apparatus after entering the leaf cells. Cd concentrations of up to 72 mg/kg (soil) had no negative effect on germination of *C. sativa*. It has been estimated from the post-conduction experiments that up to 100 ppm, there is no effect of cadmium metal on the morphological growth of *C. sativa*. The highest concentration of cadmium tolerance shown by the *C. sativa* in roots is maximum 830 mg/kg and it does not affect the growth of the plant (Linger et al. 2005).

**Fig. 2:**
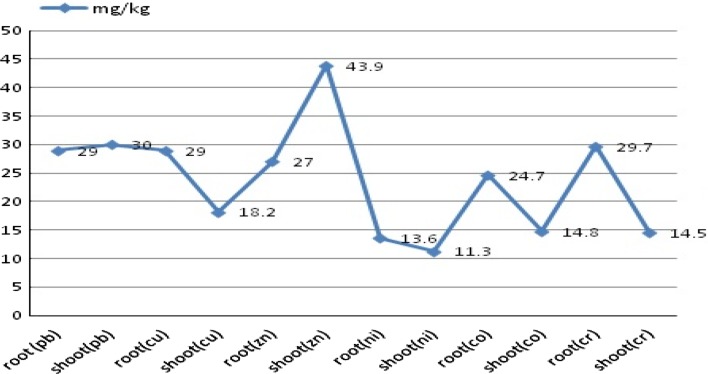
Graphical representation of accumulation of toxic heavy metals by *Cannabis sativa* (Malik et al. 2010)

The plant viability and vitality is affected by cadmium metal in the leaves and stem of *C. sativa* and was up to 50–100 mg Cd/kg [dry mass (d.m.)]. Control plants and plants growing on soil with 17 mg Cd/kg of soil show seasonal changes in phytosynthetic performance. Under moderate cadmium concentrations, i.e., 17 mg Cd/kg of soil, Hemp could preserve growth as well as the photosynthesis apparatus, and long-term acclimatization at chronicle levels to Cd stress occurs. Growth on high Cd concentrations, i.e., above 800 mg/kg leads to a significant loss of vitality and biomass production. Shi et al. (2012) worked on 18 Hemp accessions to screen the accessions that can be cultivated in cadmium (Cd)-contaminated soils for biodiesel production. Pot experiments were carried out to evaluate the ability of Hemp for Cd tolerance and bioaccumulation when subjected to 25 mg Cd/kg [dry weight (DW)] soil condition, in terms of plant growth, pigment contents, chlorophyll fluorescence and Cd accumulation at 45 days after seedling emergence. Pot experiment analysis were carried out and it was observed that most of the Hemp except USO-31, Shenyang and Shengmu, could grow quite well under 25 mg Cd/kg (DW) soil condition. A biomass of >0.5 g/plant, high tolerance factor (68.6–92.3 %) and a little reduction in pigment content and chlorophyll fluorescence under 25 mg Cd/kg (DW) soil stress were observed in Yunma 1, Yunma 2, Yunma 3, Yunma 4, Qujing, Longxi, Lu’an, Xingtai, and Shuyang. The scientist concluded that these cultivars could be cultivated in Cd-contaminated soils and had a strong tolerance to Cd stress (Shi et al. 2012). Hemp has been found to be highly cadmium-tolerant and very useful in bioaccumulation of cadmium with its superior ability to accumulate cadmium in shoots. Hemp does have a high capacity for phytostabilization. Hemp is tolerant to contaminants, has the ability to accumulate metals along with stabilization of contaminated areas and, unlike most plants used in bioremediation, it offers additional end uses. The extraction capability for heavy metals from the soil makes the Hemp (*C. sativa*) an excellent soil phytoremediation agent. Worldwide, Hemp can provide both an economic and sustainable solution to the contamination of soils. The utilization of various supplements of Hemp as a derived food has brought attention to the potential negative effects that could be caused due to potential metal accumulation on the health of people of Romania reported in the recent years (Bona et al. 2007; Linger et al. 2002; Mihoc et al. 2012). The work done on the translocation rate of certain species showed that certain species have high translocation rate as compared to other. The work concluded by Malik et al. (2010) shows that *C. sativa* has high translocation rate as compared to other species for the metal Zn and could be used as a potential hyperaccumulator for the metal Zn. It is estimated that the translocation factor value of *C. sativa* for Zn is >1. Due to this, the accumulation of metal Zn in shoots is high as compared to other heavy metals. The graph in the Fig. 2 shows the hyperaccumulation by *C. sativa* in its various tissues from contaminated soil having different heavy metals in elevated state. Investigation of metalliferous tissue of *C. sativa* leads that accumulation of zinc metal occurs maximum in shoots and it shows hyperaccumulating property by storing metal in their shoot. The least metal accumulated by *C. sativa* was Ni in their shoot (Table 2). In another work it was reported that the geochemical characterization of soil and the nature of crops are the factors on which the accumulation of heavy metals depends; some of them have a high potential to accumulate higher concentrations of heavy metals (Linger et al. 2002). The researchers investigated and concluded that one of the Hemp variety Zenit shows high bioaccumulation rate for iron, i.e., 1,859 (mg/kg) as compared to the other Hemp varieties, i.e., Diana, Denise, Armanca, and Silvana (Mihoc et al. 2012) and this could possibly effect the health of people. This work brings into light the capability of specific *Cannabis* varieties for their extraction capability.

**Table 2:** Hyperaccumulatory nature of *Cannabis sativa* is shown by the accumulation of various metals (mg/kg) (d.m.) in industrial areas (Malik et al. 2010)

Concentration of metal (mg/kg)	Root	Shoot
Lead	29 mg/kg	30 mg/kg
Copper	29 mg/kg	18.2 mg/kg
Zinc	27 mg/kg	43.9 kg/kg
Nickel	13.6 kg/mg	11.3 mg/kg
Cobalt	24.7 mg/kg	14.8 mg/kg
Chromium	29.7 mg/kg	14.5 mg/kg

### *Solanum nigrum* general properties

The common name of *Solanum* nigrum is black nightshade. It belongs to the *Solanaceae* family. *S.nigrum* is widely used plant in ornamental medicine. *S. nigrum* L. is an annual herb 0.3–1.0 m in height (Wei et al. 2005). It is anti-tumorigenic, antioxidant, anti-inflammatory, hepatoprotective, diuretic and antipyretic herb. Compounds present in the *S. nigrum* are responsible for diverse activities. Ailments such as pain, inflammation and fever are treated with *S. nigrum* as traditionally acceptable method (Zakaria et al. 2006; Lee and Lim 2003; Raju et al. 2003). The flowering season of *S. nigrum* is from July to September, and the seeds ripen from August to October. Herbal medication studies have proved that growth of cervical carcinoma in mice can be inhibited by this weed (Jian et al. 2008). Black nightshade is a fairly common herb, found in many wooded areas. *S. nigrum* grows in damp shady spots (contaminated ground) and in wastelands (Wei et al. 2005). It also grows in cultivated lands. It is a native to Europe and Asia, and further has been introduced in America, Australia and Africa through anthropogenic sources.

### Hyperaccumulative action by *S. nigrum*

*Solanum* nigrum, a newly found hyperaccumulator, has shown the effect of cadmium toxicity on the nitrogen metabolism in their leaves (Wang et al. 2007). Cadmium is very hazardous to human health adversely affecting kidney and lungs. The activity of nitrate reductase in plants is inhibited due to the effect of cadmium on the uptake and transportation of nitrates by effecting the nitrate assimilation (Hernandez et al. 1997). *S. nigrum* could tolerate ≤12 mg/kg cadmium present in the soil and maintain N metabolism normal in the plant. However, N metabolism is severely inhibited at level of 48 mg Cd/kg. The nitrate reductase activity reduces significantly at 24 mg/kg, but the activities of glutamine synthetase remains normal. *S. nigrum* appears to be an adequate species for phytoremediation of heavy metal contamination, and especially Cd contamination and shows the hyperaccumulating properties. The effect of addition of different fertilizers on the phytoremediation capability of *S. nigrum* was investigated in a study conducted by Wei et al. (2012). Under pot-culture system, these experiments were carried out by fertilizer addition, which increased the phytoextraction efficiencies of *S. nigrum* to Cd by increasing its shoot biomass. It was found that addition of chicken manure decreased the Cd concentrations of *S. nigrum*, but urea amendment did not affect organ Cd concentration. Considering the effect of decrease of available Cd in soil occurring by chicken manure, urea might be a better fertilizer for strengthening phytoextraction rate of *S. nigrum* to Cd, and chicken manure could be a better fertilizer for phytostabilization.

Zn is a micronutrient for plants, at higher concentrations it may become toxic (Broker et al. 1998; Ebbs and Cochin 1997). The Zn accumulation by *S. nigrum* decreases, when manure or compost are added up to 80 and 40 % but there will be an increase in the total biomass yield (Marques et al. 2008a).

*S. nigrum* in its efforts to remediate the metal-contaminated soil is supported by arbuscular mycorrhizal fungi (AMF). AMF occur in the soil of most ecosystems, including polluted soils. The unique structures, such as vesicles and arbuscules, are formed by the fungi belonging to the phylum Glomeromycota (Burdett 2002). The wild weeds tolerance to biotic and abiotic stresses is enhanced by the presence of AMF in the roots of the weeds present in contaminated area, as they provide a direct link between soil and roots (Joner and Leyval 1997). The availability of AMF in the roots increases the hyperaccumulation of metals by the plant. AMF are important root symbionts and partners that help in the removal of contaminants from the soil (Merharg and Cairney 2000). The fungal hyphae can extend into the soil and uptake large amounts of nutrients, including metals, to the host root. Plants provide important compounds for AMF survival; these fungi expand the contact surface between plants and soil, contributing to an enhanced plant uptake of macronutrients (Li et al. 1991) such as Zn (Burkert and Robson 1994). AMF helps plants, adapt to metal-contaminated soils, either by excluding the metals or enhancing their uptake by the plant. AMF are found everywhere in most terrestrial ecosystem, forming close or symbiotic associations with the roots of the majority of plant (Smith and Read 1997). With the help of extended properties of metal absorption from soil through AMF, *S. nigrum* becomes highly capacitive for overall extraction of metal from contaminated soil. Apart from high absorption capacities, it also has sufficient accumulation capability and high translocation property which make *S. nigrum* an ideal tool for phytoremediation approach of metal-contaminated sites. Significantly higher level of zinc metal has been observed by workers (Marques et al.2008a, b) in the *S. nigrum* supporting its hyperaccumulatory behavior. A good candidate for phytoremediation strategy would be a species that has good translocation of the metallic contaminant from the root to the stems and leaves, which means a higher translocation factor. High translocation factors (TF <1) obtained indicates that *S. nigrum* might be a good Zn phytoextractor, as the main metal accumulation occurs in the aboveground part of the plant.

A set of studies were carried out at Shenyang Zhangshi irrigation area, which was polluted with high amount of cadmium. *S. nigrum* was used in situ as a phytoremediator in Cd-polluted soil (Ji et al. 2011). Assessment of the performance of the plant over the whole growth stage was carried out. It was analyzed through experiments that aboveground biomass of single *S. nigrum* L. grew by a factor of 190, from 1.6 ± 0.4 to 300.3 ± 30.2 g along with 141.2 times extractable Cd increase from 0.025 ± 0.001 to 3.53 ± 0.16 mg. The data analysis also showed that the percentage of biomass and extracted Cd in the stem increases from 20–80 to 11–69 %, respectively. After pot experimentations, analysis was carried out for highest Cd concentration in each part of *S. nigrum* plant and observed that at seedling stage the above ground biomass was 16.1 ± 1.1 mg/kg, in stem it was observed as 12.4 ± 1.1 mg/kg and in leaf the values were 24.8 ± 2.4 mg/kg. The authors suggest that the results of their work provide reference values for the future research on the application of *S. nigrum* L. in phytoremediation or on chemical, agricultural strategies for phytoextraction efficiency enhancement. The pot experimentation establishes *S. nigrum* as a Cd hyperaccumulator with a maximum concentration of 125 mg/kg (Wei et al. 2005).

**Fig. 3:**
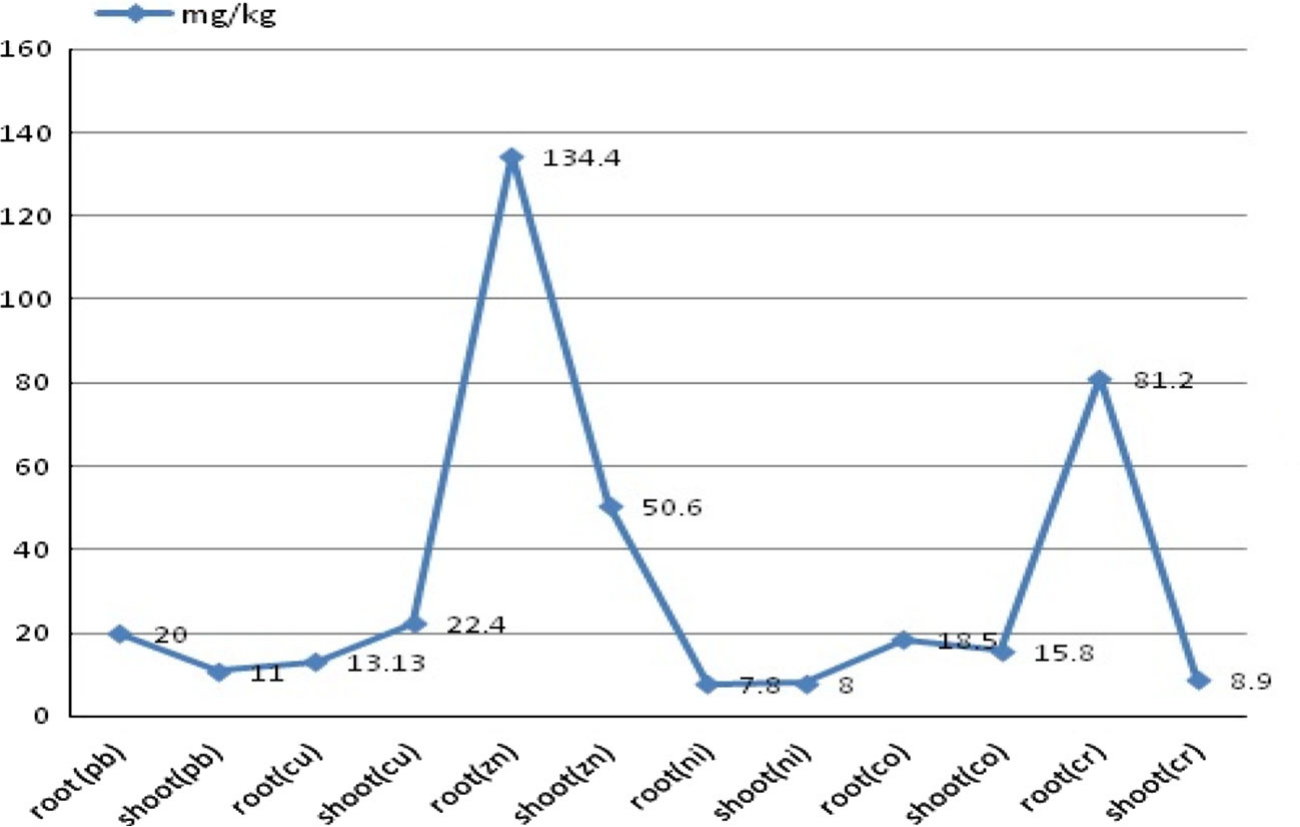
Graphical representation of accumulation of toxic heavy metals by *Solanum nigrum* (Malik et al. 2010)

The graph describes the hyperaccumulating nature of *S. nigrum* in metal-contaminated soil (Fig. 3). Significant Zn metal accumulation occurs in the root tissue of *S. nigrum*, and minimum accumulation is reported in their shoot tissue (Table 3).

**Table 3:** Hyperaccumulatory nature of *S. nigrum* shown by the accumulation of various metals (mg/kg) (d.m.) in industrial areas (Malik et al. 2010)

Concentration of metal (mg/kg)	Root	Shoot
Lead	20 mg/kg	11 mg/kg
Copper	13.13 mg/kg	22.2 mg/kg
Zinc	134.4 mg/kg	50.6 kg/kg
Nickel	7.8 kg/mg	8 mg/kg
Cobalt	18.5 mg/kg	15.8 mg/kg
Chromium	81.2 mg/kg	8.9 mg/kg

### *Rorippa globosa* general properties

*R. globosa* is an annual/perennial herb belonging to the Brassicaceae family (mustard family) and the genus *Rorippa* (yellowcress), which grows to a height of 0.7 m (2 ft 4 in.). It is a flowering plant, usually with cross shape, yellow flowers and peppery flavor. The flowering season of *R. globosa* is from April to November. The *R. globosa* is widely distributed in Europe through Central Asia, Africa, and North America. The habitat of this species is near river banks, moist areas, grasslands, and railroad embankments from near sea level to 2,500 m (Flora of China Editorial Committee 2001).

### Hyperaccumulative action by *Rorippa globosa*

Phytoremediation-based studies work on the motive for screening out and breeding hyperaccumulative plants or hyperaccumulators that have an innate capacity to absorb and accumulate metals at higher levels (Baker and Brooks 1989a, b). The leaves of *R. globosa* show no phytotoxicity or biomass reduction when exposed to 25 μg Cd/g, and the concentration of cadmium accumulated in leaves was up to 218.9 μg Cd/g (DW). Analysis of this study points toward strong self-protection ability of *R. globosa* toward cadmium metal by adapting oxidative stress caused by the cadmium exposure (Sun et al. 2010). An attractive feature of *R. globosa* provides its plantation capability twice in a year in metal-contaminated soils. *R. globosa* can be harvested at its flowering phase based on the site climatic conditions and growth characteristics of the hyperaccumulator. This could result with an increase in the extraction efficiency of Cd in shoots of *R. globosa* by 42.8 % as compared to, its single maturity state when the plant was transplanted into contaminated soils (Wei and Zhou 2006).

A comparative assessment of hyperaccumulative capability of two different species of *Rorippa* was carried out by Wei and Twardowska (2013). Six Cd treatments were experimentally designed and treatment was given to two species *R. globosa* and *R. palustris*. Different concentrations of Cd were used, i.e., 2.5, 5, 10 20, and 40 mg/kg along with a control without Cd addition. The Cd hyperaccumulating properties of *R. globosa* showed that the cadmium in the aboveground organs was >100 mg/kg, with enrichment factor EF >1, translocation factor TF >1 with no significant biomass reduction at Cd doses >10 mg/kg and lack of such properties in *R. palustris*, which made these species suitable for comparative studies (Wei and Twardowska 2013). The total root lengths were decreased by 39, 41.8 and 46.3 % expressing its tolerance limitation, when Cd concentrations were 10, 20 and 40 mg/kg. In *R. palustris*, the total root lengths when compared with the control decreased by 55.3, 64.1, and 64.4 %, indicating its weak tolerance (Li et al. 2011). In comparative research analysis done by hydroponic experiments concluded that *R. globosa* showed high tolerance capability and acted as a good Cd hyperaccumulator as compared to the *R. palustris*.

Recent studies show that the growth of *R. globosa* is skewed by the antagonist effect caused by the Cd and As metal exposure. It was observed that, when the concentration of Cd in the soil was 10 mg/kg and the concentration of As was 50 mg/kg, the plant grows up to a height of 35.9 cm and the dry weight of the shoots reaches up to 2.2 g/pot (Wei et al. 2005). At the same time, the accumulation of cadmium in the leaves under the combined stress of Cd and As becomes higher as compared to the same level of stress caused by the cadmium itself. At low concentration of Cd and As in the soil, the height and the shoot biomass of *R. globosa* increased, but the high concentrations of As and Cd reduce the Cd accumulation in the shoot of the plant by producing a synergic effect on the growth of the plant. Certain plants have the unique ability to transport, uptake and exclude various essential or non-essential metals through their roots (Fayiga et al. 2004). Exclusion and accumulation are the two main strategies that are essential to build a relationship between roots and metals. From the above study, another attractive feature of *R. globosa* comes into the picture, it has the ability to accumulate Cd metal into their various body parts such as stem, root, and leaves in the presence of arsenic metal, but at the same time it has the ability to exclude arsenic metal (Yang et al. 2004).

Wei and Zhou (2006) investigated Cd hyperaccumulation capacities in *R. globosa*, in pot experiments and concluded that to enhance the metal-removing efficiency in a year, the two-phase planting method can be utilized which measures the phytoextraction capability of plant by harvesting the plant at its flowering period. The biomass was 107.0 and 150.1 mg/kg of the Cd accumulation in stems and leaves, respectively, when soil Cd added was concentrated to 25.0 mg/kg. *R. globosa* when harvested at its flowering phase yielded total dry stem and leaf biomass up to 92.3 % of its full maturity, and the total cadmium concentration was up to 73.8 and 87.7 % of that at the mature phase, respectively. Climatic condition of the site and the trait of the plant growth-based factors enable *R. globosa*, so that it could be transplanted into the contaminated soils twice in 1 year, by harvesting the hyperaccumulator at its flowering phase. Following the two-phase planting method, the extraction efficiency of the plant increased by 42.8 % as compared to its single maturity state. This method of two-phase planting significantly helps in increasing the Cd hyperaccumulation in contaminated sites using the technique of phytoremediation over the course of a year.

**Fig. 4:**
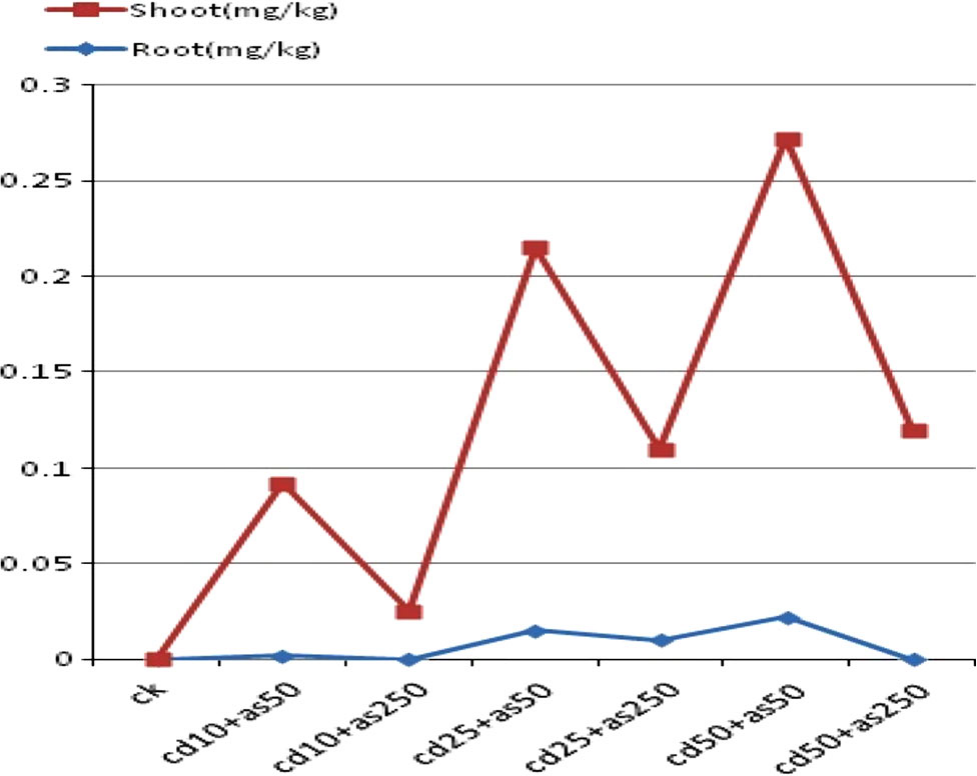
Graphical representation of antagonistic effect by Cd and As on the bioaccumulation potential of Cd hyperaccumulator *R. globosa* (Sun et al. 2007)

It can be concluded that, Cd hyperaccumulator has the basic characteristics of weed plants and the benign feature of crops. Because of the unique features such as nutrition-competitive ability, fast growth, high efficiency of photosynthesis, a short lifecycle and anti-pests capability, *R. globosa* has incomparable advantages compared with other hyperaccumulators and its utilization as a potential phytoremediator could have substantial advantages (Sun et al. 2007) (Table 4; Fig. 4).

**Table 4:** Representation of the antagonistic effect caused by the Cd and As on the bioaccumulation potential of Cd hyperaccumulator *R. globosa* (Sun et al. 2007)

Concentration of Cd and As (mg/kg)	Accumulation of Cd (mg/kg) in root	Accumulation of Cd (mg/kg) in shoot
Ck (control)	0	0
Cd10 + As50	0.002	0.09
Cd10 + As250	0	0.025
Cd25 + As50	0.015	0.2
Cd25 + As250	0.01	0.1
Cd50 + As50	0.022	0.25
Cd50 + As250	0	0.12

## Conclusion

Phytoremediation is emerging and a promising bio-based technique, which is a low-cost alternative as compared to chemical-based technologies in the clean up of heavy metal-contaminated soils. In current works, advancement is going on in various aspects of phytoremediation and for the absorption of heavy metals, where basic process understanding, responsible for the remediation processes needs to be addressed. The potential activity of wild weeds growing in metal-contaminated soil accumulates metals by showing their hyperaccumulation characteristics, and hence proving their phytoremediation mettle. In the *S. nigrum,* AMF play an important role in the accumulation of zinc metal. Addition of different fertilizers including chicken manure also plays an important role in stabilization and extraction protocols. *C. sativa* also shows the hyperaccumulation nature by accumulating cadmium metal. *R. globosa* also shows Cd hyperaccumulation characteristic by showing a unique property of antagonistic effect on the growth and the biomass concentration in the presence of arsenic metal. Two-phase planting methods have also been proposed in case of *R. globosa* for effective accumulation and extraction-based phytoremediation protocols for cadmium metal. The hyperaccumulating nature of plants depends on the type of species, soil quality, and its inherent control. All the weeds undertaken in the current study are capable of sufficient level of bioaccumulation, and still they are capable of maintaining their growth rates and reproduction levels as compared to controls in studies undertaken. Certain number of pilot scale studies should be planned by researchers for carrying out more analysis for finding out the capability of these weeds, so as to remove the metallic component in industrial and municipal level waste waters.
